# Flare‐Up After Maxillofacial Surgery in a Patient With Fibrodysplasia Ossificans Progressiva: An [^18^F]‐NaF PET/CT Study and a Systematic Review

**DOI:** 10.1002/jbm4.10008

**Published:** 2017-07-05

**Authors:** E Marelise W Eekhoff, J Coen Netelenbos, Pim de Graaf, Max Hoebink, Nathalie Bravenboer, Dimitra Micha, Gerard Pals, Teun J de Vries, Adriaan A Lammertsma, Pieter GHM Raijmakers, Robert JJ van Es

**Affiliations:** ^1^ Department of Internal Medicine Section Endocrinology VU University Medical Center Amsterdam The Netherlands; ^2^ Department of Radiology & Nuclear Medicine VU University Medical Center Amsterdam The Netherlands; ^3^ Department of Clinical Genetics and Bone Histomorphology VU University Medical Center Amsterdam The Netherlands; ^4^ Department Periodontology Academic Centre for Dentistry Amsterdam (ACTA) University of Amsterdam and VU University Amsterdam The Netherlands; ^5^ Department of Oral and Maxillofacial Surgery Utrecht University Medical Center Utrecht The Netherlands

**Keywords:** FIBRODYSPLASIA OSSIFICANS PROGRESSIVA, FOP, TRISMUS, MAXILLOFACIAL SURGERY, HETEROTOPIC OSSIFICATION, HO, [^18^F]‐NAF PET/CT, SYSTEMATIC REVIEW, FLARE‐UP

## Abstract

Fibrodysplasia ossificans progressiva (FOP) is a rare genetic disorder leading to progressive heterotopic ossifications (HO) of muscles, tendons, and ligaments, which can be induced by trauma or by surgery. Despite strong medical advice to the contrary, an FOP patient insisted on surgery to alleviate her complete trismus, which caused an unbearable impact on her quality of life (QOL). The entire trismus history of this FOP patient is presented. [^18^F]‐NaF position emission tomography/computed tomography (PET/CT) scans were introduced as an imaging method for heterotopic bone formation activity. To place our findings into context, a systematic review on jaw surgery in FOP was performed. After falling down the stairs, a 9‐year‐old patient developed mobility impairment of her left‐sided jaw. During the following 13 years bone scintigraphy showed persistent activity of the disease leading to progressive left‐sided zygomatico‐mandibular fusion by HO, resulting in complete trismus. Within 1 month after HO removal on the left side and a matching right coronoidectomy, [^18^F]‐NaF PET/CT demonstrated a substantial flare‐up activity followed by new HO in both masseter and temporalis muscles. Despite recurrent HO and trismus her QOL increased due to a stable increased interincisal opening of 5.5 mm. Although systematic review reveals a 100% risk of HO recurrence after jaw surgery, information on improved QOL is scarce. In conclusion, surgery in FOP may be beneficial for QOL despite new HO formation. Assessment of disease activity using [^18^F]‐NaF PET/CT is possible before HO is evident on CT and may serve as a new and quantitative marker of the disease. © 2017 The Authors. *JBMR Plus* Published by Wiley Periodicals, Inc. on behalf of the American Society for Bone and Mineral Research.

## Introduction

Fibrodysplasia ossificans progressiva (FOP) is a rare genetic disorder causing progressive heterotopic ossification (HO) of muscles, tendons, and ligaments.^1^, ^2^ The activating R206H mutation of the bone morphogenetic protein (BMP) type 1 receptor ACVR1 accounts for most of the classical FOP cases.^3^, ^4^ From early childhood onward, the disease becomes gradually worse owing to active periods (flare‐ups), which may be spontaneous or induced by trauma or surgery.^5^ The underlying mechanism of flare‐ups is complex and not well understood, no validated biomarkers of flare‐ups exist. One early complication of HO is represented by trismus, or restricted jaw opening, which occurs in 70% of patients at a mean age of 19 years and restricts quality of life (QOL) by interfering with essential functions such as eating, vomiting, and oral hygiene.^6^, ^7^ There is no proven treatment for FOP and surgery is contraindicated.^8^

This work presents the medical history and QOL of an FOP patient, who developed a complete trismus and insisted on maxillofacial surgery to augment her interincisal distance (ID) while accepting the risks of recurrent HO.

For the first time ^18^F‐sodiumfluoride position emission tomography/computed tomography ([^18^F]‐NaF PET/CT) scans were performed as a potential marker of flare‐ups.^9^ Results are placed into context by relating them to the outcome of a systematic review on the impact of maxillofacial surgery in FOP.

## Methods

### [^18^F]‐NaF PET/CT

[^18^F]‐NaF PET/CT scans were performed at 1, 6, and 12 months after surgery. Sixty minutes after injection of 1.2 MBq · kg^−1^ [^18^F]‐NaF, whole‐body PET images were acquired using a Gemini TF‐64 PET/CT scanner (Philips Medical Systems, Best, The Netherlands). Low‐dose CT images (30 mAs) were used for attenuation correction. ^18^F activity in regions of interest (ROIs) was expressed as standardized uptake value (SUV)^10^ reflecting early mineralization. HO was expressed in Hounsfield units (HU) derived from the CT images. SUV and HU values from ROIs were compared with reference areas of normal gluteus muscle and of the mandibular symphyseal bone.

### Systematic review

A literature search on maxillofacial surgery in FOP was performed in Medline, EMBASE, and Cochrane Library databases from inception until the middle of September 2016. The (MESH) terms and strategy used are summarized in Supporting Table  1. Nine studies on 10 case histories were included. Supporting Fig.  1 shows the inclusion process based on the Preferred Reporting Items for Systematic Reviews.

## Results

A 9‐year‐old girl developed a progressive trismus after falling down the stairs onto her face. Six and one‐half weeks later a CT scan demonstrated HO between the left mandibular coronoid process and zygomatic bone. Because of malformations of the big toes and other areas of HO, the diagnosis of classical FOP was made, which was later genetically confirmed. Repeated bone scintigraphy showed increased activity in the left inner parazygomatic region, extending to the left inner paramaxillary region, with slow increase of local HO without signs of clinical flare‐up during the following 13 years.

Six years after the fall the patient was completely unable to open her jaw, which seriously compromised her social and essential functions. She urgently requested for surgery, which was initially declined in line with the international expert advice. After 13.9 years she developed a chronic pericoronitis of her lower wisdom teeth, which required their removal. At this point she had a debilitating fear of nausea and choking resulting in frequent visits to the emergency department. After careful considerations, her request for surgery was accepted by a maxillofacial surgeon (RvE) experienced in treatment of craniofacial malformations. Under general anesthesia using fiber‐optic nasoendotracheal intubation, ostectomy of the left‐sided zygomatico‐mandibular bony fusion was executed and the buccal fat pad was interposed. Because ID only increased to 24 mm, right coronoidectomy was performed, leading to an ID of 35 mm immediately after surgery. Also her four wisdom teeth were removed.

Preoperatively, the patient received 100 mg prednisolone intravenously, which was continued orally with a dose of 100 mg per day for 1 week in combination with a nonsteroidal anti‐inflammatory drug and prophylactic antibiotic therapy. One week postoperatively the ID already had decreased to 21 mm. Two weeks after the operation, she started frequent exercises with a Therabite (Atos Medical Inc., West Allis, WI, USA), a hand‐operated device that aims to restore jaw mobility. Prednisolone treatment was slowly tapered off, but was repeated three times during the following 4.3 months as a consequence of clinical flare‐ups and related imaging results.

**Figure 1:**
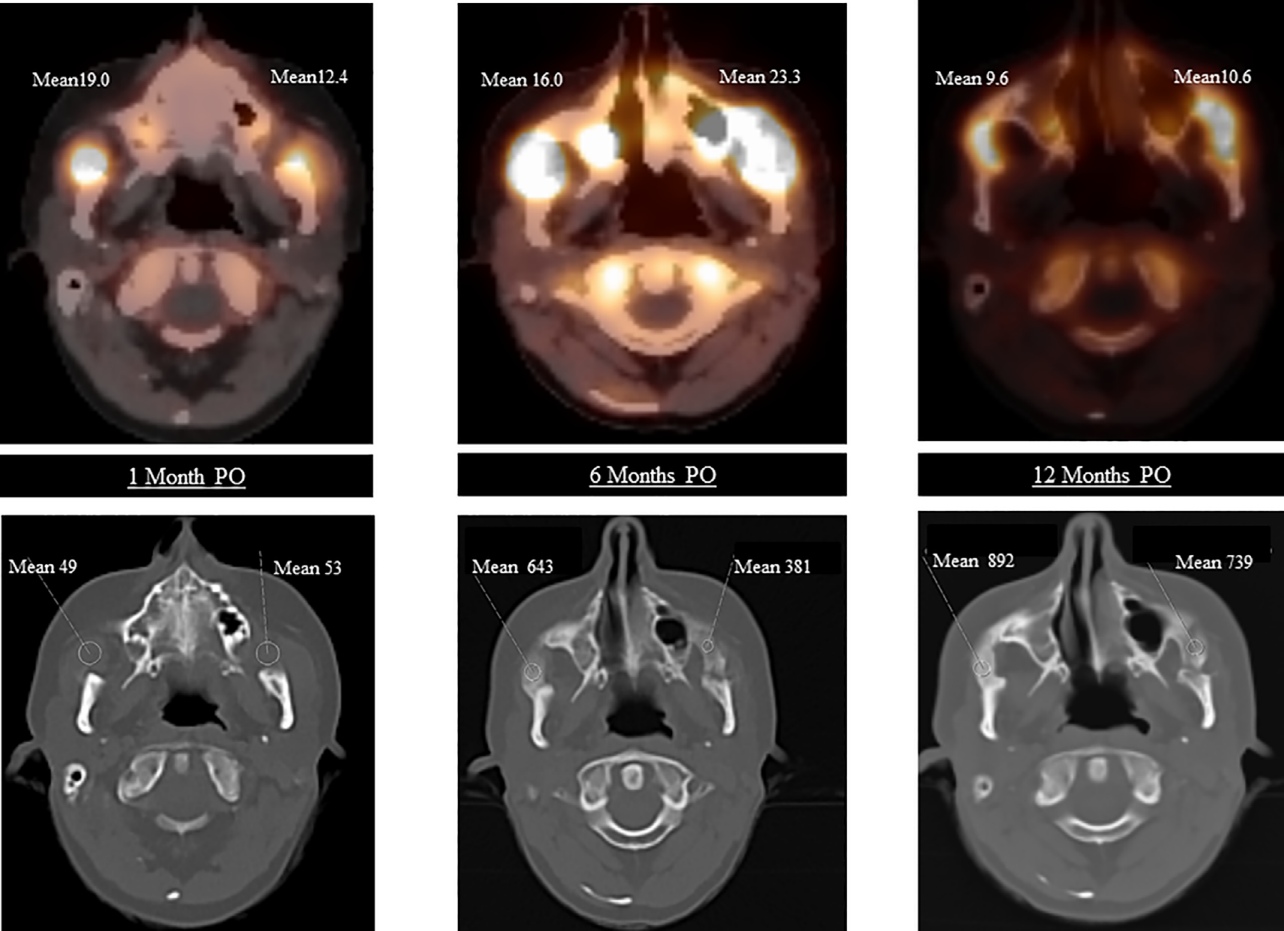
Axial [^18^F]‐NaF PET/CT images of the skull at (*A*) 1 month, (*B*) 6 months, and (*C*) 12 months PO. Upper panels show increased uptake of [^18^F]‐NaF as indicated by means of SUV. Lower panels show possibly HO after 1 month, increasing HO at 6 and 12 months as indicated by means in HU. PO = postoperatively; SUV = standardized uptake value; HO = heterotopic ossification; HU = Hounsfield units.

**Figure 2:**
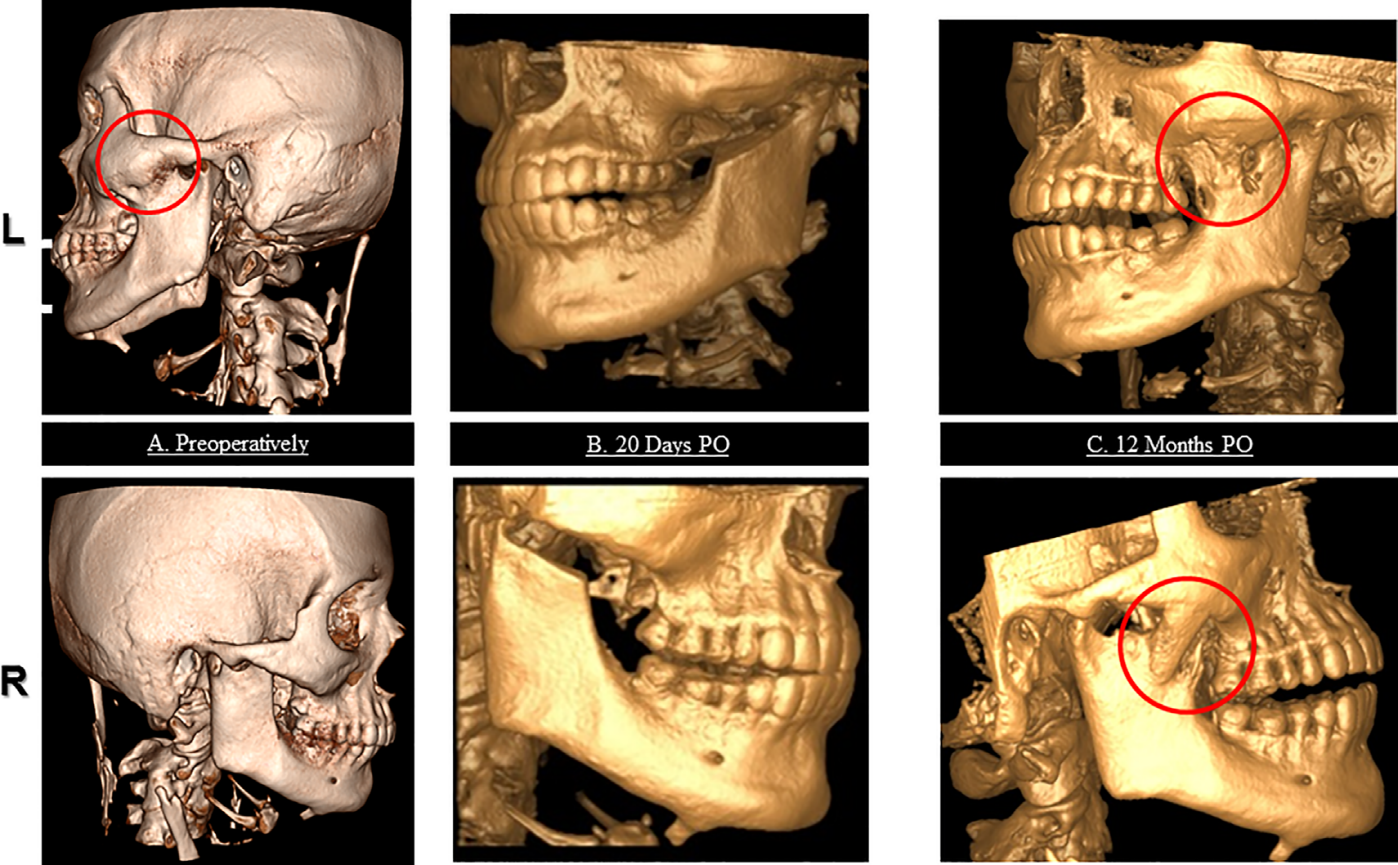
3D‐CT scan reconstruction of the skull in left (upper panel) and right (lower panel) lateral view before bilateral coronoidectomy (*A*), 20 days (*B*), and 12 months (*C*) PO. Heterotopic lesions are marked by red circles. PO = postoperatively.

Subsequent [^18^F]‐NaF PET/CT scans 1, 6, and 12 months after surgery, showed local increased muscle ^18^F uptake on both sides of the masseter and the temporalis muscle regions. After 1 year only new HO had been formed at these locations, resulting in a bony fusion of the mandibular ramus with the zygomatic bone as depicted in Figs. 1 *A*–*C* and 2 *A*–*C*. Table 1 summarizes the main imaging results at follow‐up.

**Table 1:** Results of [^18^F]‐NaF PET/CT After Maxillofacial Surgery

Indices postsurgery	Left masseter muscle region	Right masseter muscle region
Mean SUV		
Ref.: SUV gluteus muscle	0.7	0.7
After 1 month	12.4	19.0
After 6 months	23.3	16.2
After 12 months	10.6	9.6
AUC 1 year	190.95	165.67
Mean HU		
Ref.: HU over mandibular bone	1450	1450
After 1 month	53	49
After 6 months	381	643
After 12 months	739	892

SUV = standardized uptake value of ^18^F of ^18^F‐NaF PET/scan; Ref. = reference value not affected area; AUC = area under the curve; HU = Hounsfield unit of measured CT area of ^18^F‐NaF PET/scan.

Despite renewed trismus there was stable increased ID, of +5.5 mm 2 years after surgery. One year after surgery the patient felt no fear for vomiting and was no longer depressed. She had improved oral hygiene and regained an emotionally stable and socially active life. A slight anterior open bite was cosmetically acceptable. The patient rated her overall well‐being improved with an increase of six points on a well‐being visual analogue scale of 0 to 10.

Notably, no changes in inflammatory or bone‐related parameters were noticed in the laboratory analyses during the flare‐up as summarized in Supporting Table  2.

The systematic review revealed a recurrent HO with trismus after maxillofacial surgery in all 10 published case reports. No therapy or surgical procedure could prevent this (Supporting Table  3). Mean surgical follow‐up was 2.2 years (range, 0.8 to 14 years). Postoperatively the ID decreased in all but one patient.

## Discussion

This study confirms that maxillofacial surgery and removal of HO in a patient with FOP results in a flare‐up, leading to recurrent and new HO in the region, where surgery was performed. However, despite renewed trismus, the QOL of this patient improved remarkably due to the net increased ID.

A systematic review of the literature supports the findings in this case and suggests a 100% risk of recurrent HO after maxillofacial surgery with or without further intervention. Only one case report addressed the improvement in well‐being and reported a similar reduction of trismus.^11^ Following removal of HO from other sites of the body a recurrence rate of HO of 70% to 90% has been reported.^7^, ^11^

Early detection of the postoperative flare‐up was possible using [^18^F]‐NaF PET/CT. Previously, ^99m^Technetium bone scintigraphy has been used in FOP for only diagnostic purposes.^8^ [^18^F]‐NaF PET/CT has the advantage that it combines the high sensitivity of PET with precise CT localization. In [^18^F]‐NaF PET/CT, the exchange of the ^18^F ions with hydroxyl groups in the hydroxyapatite crystal of bone [Ca_10_(PO_4_)_6_OH_2_] during the process of mineralization reflects osteoblast activity, which allows for quantification of bone formation in vivo.^10^, ^12^ This technique has been validated in studies showing a correlation between ^18^F uptake and bone histomorphometric findings.^12^, ^13^ [^18^F]‐NaF PET/CT shows increased physiological bone formation within 2 weeks after allograft replacement in healthy persons.^9^ In the present case markedly increased fluoride uptake in muscles was shown already at the first [^18^F]‐NaF PET/CT scan 1 month after surgery. New HO only developed within muscle regions where increased ^18^F activity was seen, predicting the location of HO formation at an early stage. To the best of our knowledge this is the first time FOP disease activity was demonstrated during a defined flare‐up. With the aid of this new diagnostic tool, the pathophysiology of mysterious flare‐ups in FOP may become better understood.

In conclusion, maxillofacial surgery in FOP can be beneficial for the patient despite exacerbation of the disease, but should remain a management choice of last resort. [^18^F]‐NaF PET/CT is a promising technique to characterize and quantify flare‐ups in FOP, and therefore could become a new tool in proof‐of‐concept trials to evaluate novel drugs designed to prevent HO.

## Disclosures

All authors state that they have no conflicts of interest.

## Supporting information

Supporting Data S1.
