# Supplementary material for: Flare‐Up After Maxillofacial Surgery in a Patient With Fibrodysplasia Ossificans Progressiva: An [18F]‐NaF PET/CT Study and a Systematic Review
Source: JBMR Plus. 2017 Jul 5;2(1):55–8. doi: 10.1002/jbm4.10008 (PMC6124206; doi:10.1002/jbm4.10008)
Supplement: Supplementary file 1 — Supporting Data S1. [file JBM4-2-55-s001.pdf]

Supplementary data related to the manuscript entitled:

Flare-up after Maxillofacial Surgery in a Patient with Fibrodysplasia Ossificans Progressiva: an [^{18}F]-NaF PET/CT Study and a Systematic Review

E. Marelise W. Eekhoff,[1] J. Coen Netelenbos,[1] Pim de Graaf,[2] Max Hoebink,[1] Nathalie Bravenboer[3], Dimitra Micha,[3] Gerard Pals,[3] Teun J. de Vries,[4] Adriaan A. Lammertsma,[2] Pieter G.H.M. Raijmakers[2] and Robert J.J. van Es.[5]

*Corresponding author: E.M.W. Eekhoff, MD, PhD,

Department of Internal Medicine, Section Endocrinology, VU University Medical Center

Address: De Boelelaan 1117, 1081HV Amsterdam, The Netherlands

Emailaddress: emw.eekhoff@vumc.nl

Supplementary data included are:

Legends to supplementary Figure 1

Supplementary Tables 1, 2 and 3.

Legends Supplementary Figure 1

Supplementary Figure 1

Flow diagram of the included studies in the systematic review on FOP and maxillofacial surgery

Supplementary Figure 1

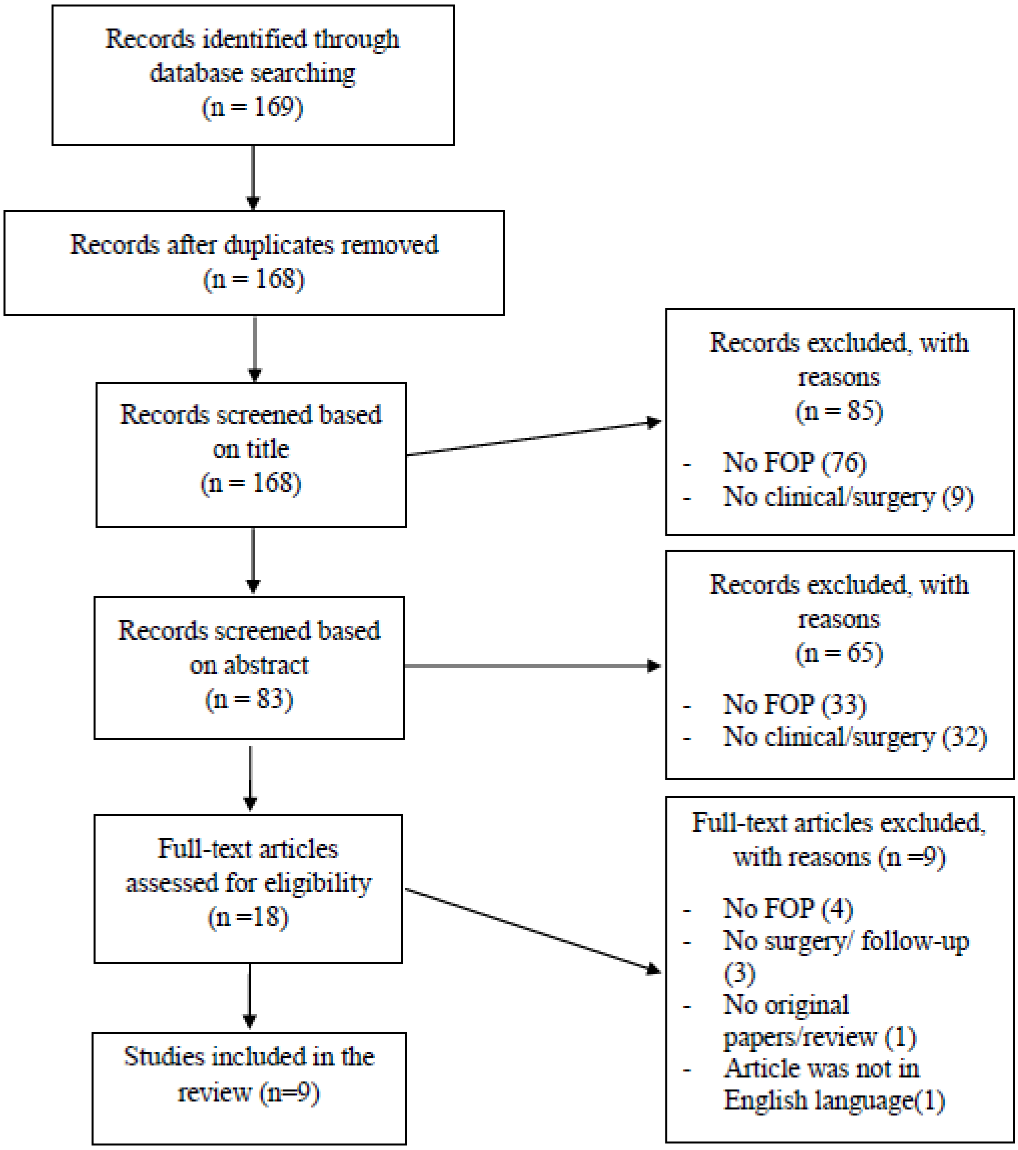

Supplementary Table 1

Key Words, (MESH) terms and Strategy used and combined for the systematic review on maxillofacial surgery in FOP

**Review Key Words**	**Mesh terms**
FOP	"Myositis Ossificans"[Mesh] OR fibrodysplasia ossificans progressiv*[tiab] OR myositis ossificans[tiab] OR stone man[tiab] OR acvr1[tiab]
Surgery	"surgery" [Subheading] OR "Surgical Procedures, Operative"[Mesh] OR "Surgeons"[Mesh] OR "Perioperative Period"[Mesh] OR "Perioperative Care"[Mesh] OR "Anesthesia"[Mesh] OR "Osteotomy"[Mesh] OR "Orthognathic Surgical Procedures"[Mesh] OR surger*[tiab] OR surgical*[tiab] OR surgeon*[tiab] OR operation*[tiab] OR operative*[tiab] OR perioperati*[tiab] OR anesthe*[tiab] OR anaesthe*[tiab] OR incisi*[tiab] OR extracti*[tiab] OR excisi*[tiab] OR ostectom*[tiab] OR osteotom*[tiab] OR coronoid*[tiab] OR invasive*[tiab] OR restorati*[tiab]
Jaw	"Facial Bones"[Mesh] OR "Tooth"[Mesh] OR "Microstomia"[Mesh] OR "Mouth"[Mesh] OR "Dental Caries"[Mesh] OR "Masticatory Muscles"[Mesh] OR "Mastication"[Mesh] OR restricted mouth[tiab] OR microstom*[tiab] OR oral[tiab] OR orally[tiab] OR masticat*[tiab] OR dental*[tiab] OR periodontal*[tiab] OR molar*[tiab] OR jaw[tiab] OR jaws[tiab] OR mandib*[tiab] OR maxill*[tiab] OR zygoma*[tiab] OR tooth[tiab] OR teeth[tiab] OR mouth*[tiab] OR locking*[tiab] OR locked[tiab] OR interocclus*[tiab] OR occlus*[tiab] OR malocclus*[tiab] OR caries[tiab] OR teeth[tiab] OR tooth[tiab] OR fusion*[tiab] OR fixati*[tiab] OR ankylos*[tiab] OR jawlock*[tiab]
**Review process**	**Procedure**
Strategy	The review process included the following strategy: excluded were articles not investigating FOP or jaw surgery. All identified titles (n=169) were independently evaluated by two reviewers (ME, CN). We selected only those studies that were related to both FOP and jaw surgery. After scrutinizing the remaining 18 articles, 9 articles were excluded because they were not related to FOP and jaw surgery, either not originally or not in the English language.

FOP= Fibrodysplasia ossificans progressiva; ME=Marelise Eekhoff; CN=Coen Netelenbos

Supplementary Table 2

Laboratory results pre- and post-maxillofacial surgery

**Blood tests**	**Pre surgery**		**Post surgery flare-up**			**No clinical flare-up**
	1 year	1 week	1 month	1.5 months	2.5 months	1 year
CRP (N<8 mg/L)	<2.5	<2.5	<2.5	8	4	
ESR (N<20mm/hr)	8					
Hb (N 7.5-10mmol/L)	8.1	7.9	7.9	7.5		
Leucocytes (N 4-10 x10^9/L	9.5	22.5	14		8.9	
MDRD (N>60 mL/min/ 1.73 m2)	>90	>90	>90	>90		
Calcium (N 2.2-2.6 mmol/L)	2.32		2.35			2.32
Phosphate (N 0.7-1.4mmol/L)	1.0					
Albumin (N 35-52 G/L)			36			37
ALP (N 0-120U/L)	57		60			56
GGT (N 0-40U/L)	10		14			6
CK (N 0-145 U/L)				49		51
Vit D (N 25-150 nmol/L)	104					
Osteocalcin (0.5-2.2 nmol/L)		1,5		1.6		
P1NP (N 19-83 µG/l)	29		22	37	39	
1CTP (N 2.1-5.6 µG/L)	4.6					
CTX (N <573 ng/L)			55			
**Urinary tests**						
Hydroxyproline (mmol/L)	0.429					0.103
Creatinine (mmol/L)	34.98					6.13

CRP=C-Reactive Protein, ESR=Erythrocyte Sedimentation Rate, Hb=hemoglobine,

MDRD= Modification of Diet in Renal Disease Study equation,

ALP= total alkaline phosphatase, CK=Creatine Kinase, Vit D= vitamin 25(OH)D,

P1NP= type 1 procollagen N-terminal, 1CTP=cross-linked carboxyterminal telopeptide of type I collagen, CTX= C-terminal telopeptide (CTX).

Supplementary Table 3

Summary of the included articles on maxillofacial surgery in FOP identified by the systematic review

**Authors**	**Gender (age in years) severity FOP**	**FOP diagnosis (age)**	**Reason for jaw surgery**	**Surgical removal**	**Follow-up time and therapy**	**Final situation (decrease of ID opening)**	**Improved jaw opening/ increased quality of life**
Connor et al.1982[1]	Female (15-34)	?	Limitation jaw movement	HO	4 months	Total occlusion	No improvement
Seguin et al. 1987[2]	? (16)	After surgery	Limitation jaw movement	HO	?	Recurrence HO	No improvement
Crofford et al. 1990[3]	Male A (21) extensive HO<br><br>Male B (16) dextroscoliosis	A (13)<br><br>B (15,5)	A: Jaw immobilization<br><br>B: ID 5 mm HO mandibular to pterygoid	A: Bridge of zygomatic arch to coronoid<br>B: HO medial pterygoid muscle	A: 8 months etidronate<br><br>B: 2 months isotretinoin and etidronate	A: ID 2 mm 1 month after operation<br>B: ID 4 mm 2 months after operation	Apparently in both patients deterioration (no improvement)
Aslan et al. 1999[4]	Female (?) with joint restrictions	?	Bilateral trismus	HO masseter muscle	1 year etidronate	Total lockjaw	No improvement
Chichareon et al 1999[5]	Male (3) torticollis	After third jaw operation (5?)	a) ID 2cm due to submandibular osteochondroma<br>b) ID 1mm by new HO bridging<br>c) ID 5 mm by recurrent HO	a) Osteochondroma (2 cm) at lower border mandibula<br>b) HO and coronoidectomy<br>c) HO after b) (anesthesia: tracheostomy)	a) 10 months physiotherapy forced opening<br>b) 5 months with physiotherapy<br>c) one month	a) ID 1 mm<br><br>b) ID 5mm<br><br>c) ID decrease rapidly within 1 month	Three operations (a, b, c) leading to deterioration with new HO cheek and neck (no improvement)

Herford et al 2003[(6)]	Male (24) wheelchair	(24)	Complete trismus for 10 years	1 cm HO between zygoma sup. and coronoid process	1 year rigorous physiotherapy	ID 15 mm	Improvement/ increased quality of life
Wadenya<br><br>et al. 2010[(7)]	Male (10)<br><br>-at 3 years diabetes type 1<br><br>-at 20 years ambulant with a cane;<br><br>-at 30 years wheelchair	Before second operation (20)	a) After trauma at age 9 progressive limited opening<br><br>b) Complete trismus after first operation<br><br>(c) Buccal approach for dental care only	a) 1st operation HO condylar head-temporal bone<br><br>b) 2nd operation bilat. subcondylar osteotomies,<br><br>c) 3rd procedure<br><br>fiberoptic bronchoscopy	a) time follow-up lost<br><br>b) after 10 years lost in follow-up<br><br>c) no follow-up	a) Complete lockjaw<br><br>b) Complete lockjaw	Two operations (a, b) leading to deterioration (no improvement)
Carvalho<br><br>et al. 2011[(8)]	Male (8)	(1)	?	Submandibular and post.neck swellings	14 years	Jaw restriction retrognatism	Apparently deterioration (no improvement)
Kriegbaum et al. 2013[(9)]	Male (26)<br><br>Only jaw involvement	after third operation (29)	ID 12 mm	a) 1st operation bony fusion mandible and zygomatic arch<br><br>b) 2nd operation bony fusion<br>c) 3rd operation preauricular incision, extensive resection of the HO	a) 1 year dilation and physiotherapy<br><br>b) salazopyrine 1 year<br>c) 6years, later etidronate and vigorous exercise	a) ID 12mm after one year<br><br>b) ID decrease thereafter<br>c) ID 8mm due to massive HO recurrence	Eventually no improvement

Reference List of supplementary Table 3

(1) Connor JM, Evans DA. Extra-articular ankylosis in fibrodysplasia ossificans progressiva. Br J Oral Surg 1982 Jun;20(2):117-21.

(2) Seguin P, Delmas P, Bouvier R, Freidel M. [Permanent constriction of the jaws due to progressive ossifying myositis]. Rev Stomatol Chir Maxillofac 1987;88(3):190-5.

(3) Crofford LJ, Brahim JS, Zasloff MA, Marini JC. Failure of surgery and isotretinoin to relieve jaw immobilization in fibrodysplasia ossificans progressiva: report of two cases. J Oral Maxillofac Surg 1990 Feb;48(2):204-8.

(4) Aslan G, Celik F, Gorgu M. Unusual ankylosis of the jaw due to fibrodysplasia ossificans progressiva. Ann Plast Surg 1999 Nov;43(5):576-8.

(5) Chichareon V, Arpornmaeklong P, Donsakul N. Fibrodysplasia ossificans progressiva and associated osteochondroma of the coronoid process in a child. Plast Reconstr Surg 1999 Apr;103(4):1238-43.

(6) Herford AS, Boyne PJ. Ankylosis of the jaw in a patient with fibrodysplasia ossificans progressiva. Oral Surg Oral Med Oral Pathol Oral Radiol Endod 2003 Dec;96(6):680-4.

(7) Wadenya R, Fulcher M, Grunwald T, Nussbaum B, Grunwald Z. A description of two surgical and anesthetic management techniques used for a patient with fibrodysplasia ossificans progressiva. Spec Care Dentist 2010 May;30(3):106-9.

(8) Carvalho DR, Farage L, Martins BJ, Speck-Martins CE. Craniofacial findings in fibrodysplasia ossificans progressiva: computerized tomography evaluation. Oral Surg Oral Med Oral Pathol Oral Radiol Endod 2011 Apr;111(4):499-502.

(9) Kriegbaum RK, Hillerup S. Fibrodysplasia ossificans progressiva (FOP): report of a case with extra-articular ankylosis of the mandible. J Craniomaxillofac Surg 2013 Dec;41(8):856-60.
